# Use of multivariate control charts to assess the status of reef fish assemblages in the Northwestern Hawaiian Islands

**DOI:** 10.7717/peerj.3651

**Published:** 2017-07-31

**Authors:** Atsuko Fukunaga, Randall K. Kosaki

**Affiliations:** 1 Joint Institute for Marine and Atmospheric Research, University of Hawai‘i at Mānoa, Honolulu, HI, United States of America; 2 Papahānaumokuākea Marine National Monument, National Ocean Service, National Oceanic and Atmospheric Administration, Honolulu, HI, United States of America

**Keywords:** Papahānaumokuākea Marine National Monument, Multivariate control chart, Assemblage structure, Fish, Ecological monitoring, Stratified random sampling

## Abstract

A distance-based multivariate control chart is a useful tool for ecological monitoring to detect changes in biological community resulting from natural or anthropogenic disturbances at permanent monitoring sites. It is based on a matrix of any distances or dissimilarities among observations obtained from species composition and abundance data, and bootstrapping techniques are used to set upper confidence bounds that trigger an alarm for further investigations. We extended the use of multivariate control charts to stratified random sampling and analyzed reef fish monitoring data collected annually on shallow (≤30 m) reefs across the Northwestern Hawaiian Islands (NWHI), part of the Papahānaumokuākea Marine National Monument. Fish assemblages in the NWHI were mostly stable, with exceptions in the south region (Nihoa, Mokumanamana and French Frigate Shoals) in 2012 and 2015 where changes in the assemblage structure exceeded the upper confidence bounds of multivariate control charts. However, these were due to changes in relative abundances of native species, and potentially related to the small numbers of survey sites and relatively low coral covers at the sites, particularly in 2015. The present study showed that multivariate control charts can be used to evaluate the status of biological communities in a very large protected area. Future monitoring of fish assemblages in the Papahānaumokuākea Marine National Monument should be accompanied by specific habitat or environmental variables that are related to potential threats to its shallow-water ecosystems. This should allow for more detailed investigations into potential causes and mechanisms of changes in fish assemblages when a multivariate control chart triggers an alarm.

## Introduction

Long-term ecological monitoring programs generally focus on investigating the current status of an ecosystem, identifying trends or detecting changes in an ecosystem caused by natural or anthropogenic disturbances (Lindenmayer & Likens, 2010). Disturbances can be classified as short-term “pulse” and longer-term “press” disturbances, although a series of pulse disturbances repeated frequently at short intervals with no time for the system to recover is considered a press disturbance (Connell, 1997). In a coral reef environment, pulse disturbances include tropical storms (Connell, Hughes & Wallace, 1997), bleaching events (Loya et al., 2001), *Acanthaster* (crown-of-thorns starfish) outbreaks (Kayal et al., 2012) and sedimentation from dredging (Brown et al., 1990), while press disturbances include urbanization and long-term sewage discharges (Hunter et al., 1995) and decline of herbivores (Bellwood et al., 2004). It is often unknown where or when these natural or anthropogenic disturbances may occur, thus requiring scientists and managers to monitor the ecosystem for an impact resulting from these disturbances.

A distance-based multivariate control chart is a useful tool for ecological monitoring to identify impacts quickly at individual sites when they occur (Anderson & Thompson, 2004). This method is based on a matrix of any distances or dissimilarities (e.g., Bray–Curtis dissimilarity, chi-squared distance, etc.) among observations obtained from species composition and abundance data. For each site, it calculates the distance between the observation at time *t* and either the centroid of the previous (*t* − 1) observations in multivariate space or the centroid of observations at time points designated as a baseline. The distance from the centroid of all previous observations (*d*_*t*_) is useful when identifying pulse disturbances, while the distance from the baseline centroid (${d}_{t}^{b}$) is sensitive to changes in species data caused by gradual press disturbances (Anderson & Thompson, 2004). For both *d*_*t*_ and ${d}_{t}^{b}$, confidence limits obtained by bootstrapping techniques are used to set upper confidence bounds, within which the system is considered to be stable or “in control”. Multivariate control charts thus trigger an alarm for further investigations when values of *d*_*t*_ and/or ${d}_{t}^{b}$ exceed these upper confidence bounds.

**Figure 1:**
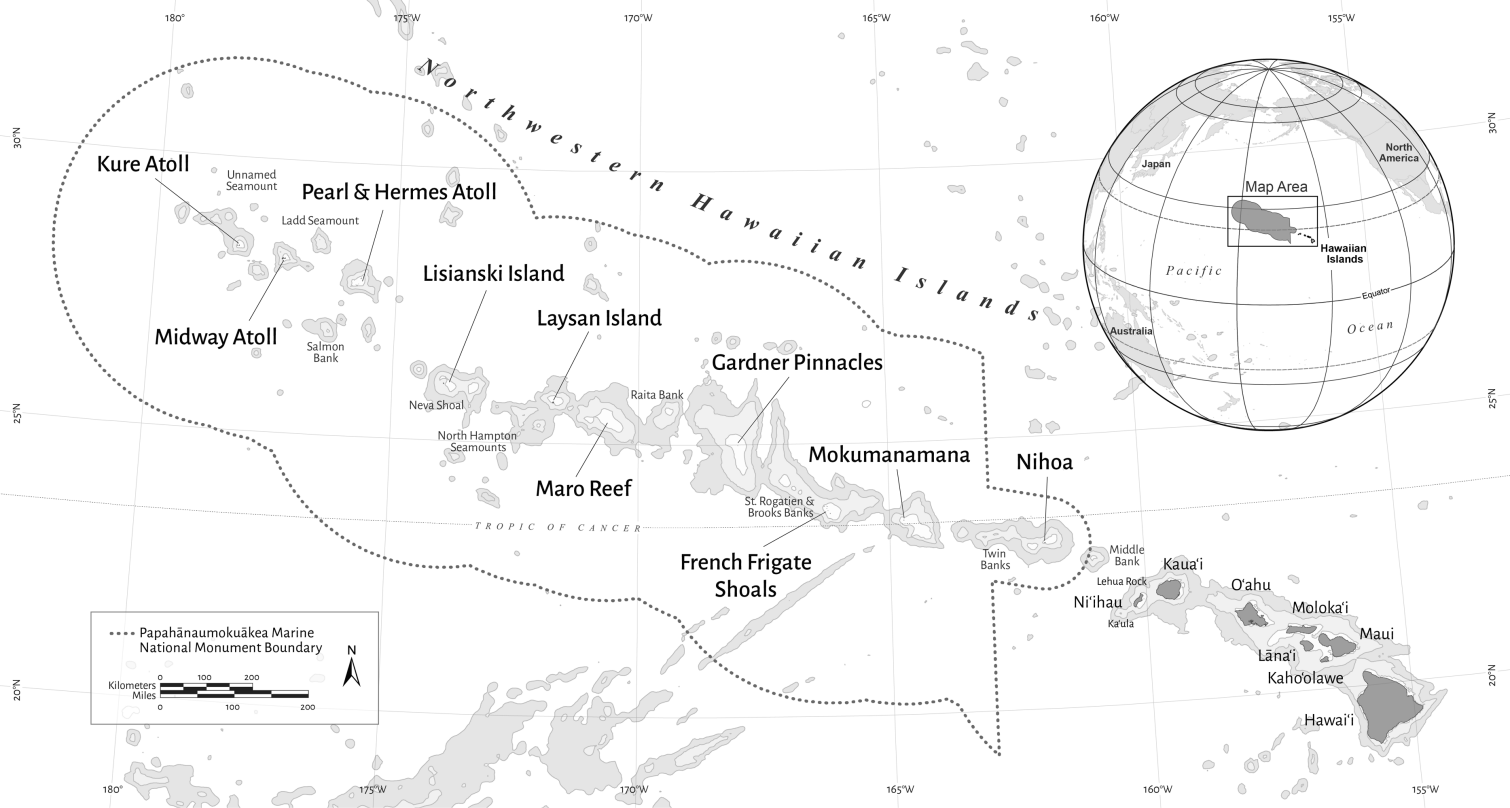
Map of the Hawaiian Archipelago including the Northwestern Hawaiian Islands (NWHI). The boundary of the Papahānaumokuākea Marine National Monument is shown by the dotted line.

The Northwestern Hawaiian Islands (NWHI) are located northwest of the inhabited main Hawaiian Islands and consist of ten major islands and atolls that span approximately 2,000 km from Nihoa (23°04′N, 161°55′W) at the southeastern end to Kure atoll (28°25′N, 178°20′W) at the northwestern end (Fig. 1). They are part of the Papahānaumokuākea Marine National Monument, a marine protected area that was originally established in 2006 and expanded in 2016 to encompass 1,508,870 km^2^; it is one of the largest conservation areas in the world. Despite their remote location and fully protected status, shallow-water reef habitats of the NWHI are still subject to natural and/or anthropogenic disturbances, such as marine debris, ship grounding, invasion of alien species (e.g., invertebrates, algae and fish) and increases in sea surface temperature due to climate change and resulting coral bleaching (Office of National Marine Sanctuaries, 2009). Monitoring of shallow-water (≤30 m) coral reef fish has been conducted annually in the NWHI during the Reef Assessment and Monitoring Program (RAMP) cruise each summer.

Here, we describe reef fish assemblages of the NWHI using the shallow-water monitoring data and demonstrate the use of distance-based multivariate control charts to detect changes in the structure of fish assemblage over time. The method of multivariate control chart was originally described using fish data collected over permanent transects at various sites at a certain time interval (Anderson & Thompson, 2004), allowing one to investigate natural temporal variability in a biological community at each site and to identify observations outside of this natural variability. However, due to the large size of the Papahānaumokuākea Marine National Monument and the wide range of reef habitats it encompasses, establishing a sufficient number of permanent transects to assess the status of shallow-water (≤30 m) habitats of the entire monument is difficult. Islands and atolls being surveyed differ every year depending on that year’s cruise schedule, so survey sites are randomly selected from survey domains (i.e., islands/atolls) stratified by reef-zone and depth range. This stratified random sampling has an advantage over permanent transects in that it allows for more precise estimates of fish population density and abundance for the entire survey domain (Smith et al., 2011) and is commonly applied to fisheries science (Cadima et al., 2005). Our method described here uses a distance-based linear model to account for variables that are part of the stratified sampling design and obtains residuals, which are, in turn, used for multivariate control charts. This method expands multivariate control charts to ecological monitoring programs where establishing permanent transects is not possible or practical.

## Materials and Methods

### Survey design

Monitoring of reef fish was annually performed using a stationary point count (SPC) method (Williams et al., 2015), a modification of the stationary visual census technique (Bohnsack & Bannerot, 1986), from 2007 to 2016, except for 2008 (surveyors used belt transects) and 2013. Surveyed islands/atolls of the NWHI (Fig. 1) were Nihoa (23°04′N, 161°55′W), Mokumanamana (23°34′N, 164°42′W), French Frigate Shoals (FFS: 23°52′N, 166°17′W), Gardner Pinnacles (Gardner: 25°01′N, 167°59′W), Maro Reef (Maro: 25°25′N, 170°35′W), Laysan Island (Laysan: 25°42′N, 171°44′W), Lisianski Island (Lisianski: 26°04′N, 173°58′W), Pearl and Hermes Atoll (PHA: 27°56′N, 175°44′W), Midway Atoll (Midway: 28°12′N, 177°21′W) and Kure Atoll (Kure: 28°25′N, 178°20′W). Survey sites at each island or atoll were chosen each year by generating random points (coordinates) on GIS bathymetric and bottom composition maps, in which target hard-bottom habitats for monitoring at depths ≤30 m were stratified by reef zone (backreef, forereef and lagoon) and depth range (shallow 0–6 m, mid 6–18 m and deep 18–30 m). Only one site each was surveyed from the backreef-mid and backreef-deep strata in the entire surveys, so these sites were omitted from analysis, resulting in seven strata in this study (Table 1): backreef-shallow (BKR-S), forereef-shallow (FRF-S), forereef-mid (FRF-M), forereef-deep (FRF-D), lagoon-shallow (LAG-S), lagoon-mid (LAG-M) and lagoon-deep (LAG-D). Nihoa, Mokumanamana, Gardner, Laysan and Lisianski do not have lagoon and backreef habitats, so shallow reefs of these locations were categorized based on depth into FRF-S, FRF-M or FRF-D.

**Table 1:** Stratification scheme for SPC fish surveys at each island/atoll.

		Depth (m)		Locations
	0–6	6–18	18–30	
Forereef	FRF-S	FRF-M	FRF-D	All islands and atolls
Lagoon	LAG-S	LAG-M	LAG-D	FFS, Maro, PHA, Midway and Kure
Backreef	BKR-S	–	–	FFS, Maro, PHA, Midway and Kure

At each site, one or two pairs of divers simultaneously performed a reef fish survey. Briefly, divers laid a 30-m transect line along a depth contour and counted fish inside adjacent SPC cylinders 15 m in diameter. For the first five minutes (species enumeration period), divers recorded all species observed in their cylinders, and during a following tallying period, they systematically worked through their lists to record the number of individuals for each species. If a species was observed during the enumeration period but absent when recording the number of individuals during the tallying period, divers recorded the number present in their cylinders when it was first observed during the enumeration period. Divers remained stationary at the centers of their cylinders to avoid disturbance as much as possible, but at the end of the tallying period they swam through their plots to count small, semi-cryptic species. Prior to participating in SPC surveys each year, all surveyors went through rigorous training for fish identification, sizing and count. Observer biases were also assessed on a daily basis throughout the cruises for quality assurance and control of survey data. In addition, starting 2010, divers performed rapid visual assessments of benthic cover by recording percent cover of major functional categories (e.g., hard corals, macroalgae and crustose coralline algae) and characterization of survey sites into reef types: aggregate reef, aggregate patch reef, aggregate patch reefs, mixed habitat, pavement, pavement with patch reefs, pavement with sand channels, rock boulder, reef rubble, spur and groove and scattered coral/rock.

Field work in the NWHI was conducted under Papahānaumokuākea Marine National Monument research permits, PMNM-2007-048, PMNM-2009-058, PMNM-2010-052, PMNM-2011-022, PMNM-2012-034, PMNM-2014-018, PMNM-2015-012 and PMNM-2016-023.

### Data analyses

Multivariate analyses of reef fish assemblages were done using the software package PRIMER 7 (Clarke & Gorley, 2015) with the PERMANOVA+ add-on (Anderson, Gorley & Clarke, 2008). Fish counts were first averaged to obtain fish abundance per survey site. Pelagic and semi-pelagic fishes, including sharks, rays, tunas, jacks, sardines, anchovies and herrings, were excluded in order to focus on non-transient, resident reef fish at each site, as the survey method was designed specifically for reef fish monitoring. Less-common species that did not account for at least 10% of the total fish abundance at any of the survey sites were also excluded in order to reduce noise in data analyses. The structure of fish assemblages as a whole was computed using the Bray–Curtis measure after square-root transformation of the abundance data.

In order to characterize fish assemblages in the NWHI, we used similarity percentage analysis (SIMPER: Clarke & Warwick, 2001) to determine fish species that typified each stratum across all years. The Bray–Curtis similarities calculated among observations within each stratum were broken down into contribution from each species. Similarity contributions of each species were then averaged. A fish species with a high average similarity contribution and a low standard deviation indicates that the species is found at a high and consistent abundance, and therefore it typifies that particular stratum. Trophic habits of the top eight fishes that typified each stratum were then determined based on various databases and references, including FishBase (ver. 06/2016, http://www.fishbase.org), the NOAA Pacific Islands Fisheries Science Center’s database (www.pifsc.noaa.gov/cred/fish.php#), Randall (2007), Hiatt & Strasburg (1960) and Hobson (1974).

Formal tests to examine relationships between the structure of fish assemblages on the basis of the Bray–Curtis dissimilarities and explanatory variables were done using a distance-based linear model (DISTLM: Legendre & Anderson, 1999; McArdle & Anderson, 2001), with 4,999 permutations of residuals under a reduced model. Due to differences in latitude, and thus water temperature in winter, reef fish assemblages in the Hawaiian Islands change along the island chain (Grigg et al., 2008). It is therefore important to account for potential effects of these geographical location, as well as strata, when examining changes in fish assemblages over time. Specifically, we used three sets of explanatory variables, location, stratum and year in the order of these variables being added to the model, and performed DISTLM conditional tests to examine the amount of additional variability explained by an explanatory variable after fitting one or more other explanatory variables, considering overlap in the variability explained by multiple explanatory variables. The rationale for this analysis was to examine the effects of habitat (i.e., stratum) on the structure of fish assemblages after large-scale spatial variability (i.e., geographical locations within the NWHI) was taken into account, and then the presence of temporal variability (year-to-year differences) after large-scale spatial variability and the effects of strata were considered. The location variable was obtained by performing principal component analysis of latitudinal and longitudinal GPS coordinates of each survey site. The first principal component (PC1) axis explained 99.5 % of the variance in the original coordinates, so centered PC1 scores were used as a single location variable. The stratum and year variables were both categorical variables with seven and eight levels, respectively.

### Multivariate control charts

For multivariate control charts, we computed the assemblage structure on the basis of the Bray–Curtis dissimilarity using the same square-root transformed abundance data, after removing pelagic and semi-pelagic fishes and less-common species. Removing less-common species could eliminate the possibility of capturing a species that was new to the NWHI in multivariate control charts unless it occurred in a relatively high number at any survey sites, but new species would have been readily noted by divers during surveys or while entering data into a database. Note that the process of removing less-common species is not required for construction of multivariate control charts, so these species may be retained if changes in their abundances are of interest. Similarly, as previously stated, multivariate control charts can be constructed using a matrix of any distances or dissimilarities (i.e., not limited to the Bray–Curtis dissimilarity), so an appropriate measure of ecological structure should be chosen based on the nature of each study.

We used regional centroids in multivariate space to calculate *d*_*t*_ and ${d}_{t}^{b}$ by dividing the NWHI into three regions based on their latitudes: south (23°N: Nihoa, Mokumanamana and FFS), mid (25–26°N: Gardner, Maro, Lisianski and Laysan) and north (27–28°N: PHA, Midway and Kure). Rationale for this use of regional (rather than island/atoll or the entire NWHI) centroids was that, at the island/atoll level, there were many missing data as none of the islands/atolls were surveyed every year, and at an archipelagic scale, it could be difficult to detect any impacts that occurred in more or less localized area. Thus, this grouping was for the purpose of detecting impacts at a spatial scale smaller than the entire NWHI, while having consistent data points for each time period, and effects of latitudinal and longitudinal differences on the structure of fish assemblages among survey sites were explicitly taken into account prior to construction of control charts (see below). Examples of potential localized effects specific to the NWHI include passing storms, severe localized bleaching (see Couch et al., 2016) and introduced fishes, particularly the snappers *Lutjanus kasmira* and *Lutjanus fulvus* and the grouper *Cephalopholis argus*, which were all intentionally introduced to the main Hawaiian Island of O‘ahu in the 1950’s and became established (see Randall, 1987).

Multivariate control charts were constructed using the statistical software R version 3.3.1 (R Core Team, 2016). In order to account for the effects of geographical location and strata on the structure of fish assemblages, we used DISTLM with the two explanatory variables, location (i.e., centered PC1 scores described above) and stratum, and obtained residuals, and these residuals are, in turn, used for multivariate control charts. Specifically, Gower’s centered matrix (**G**) was first calculated from the Bray–Curtis dissimilarity matrix among survey sites. The hat matrix (**H)** was also calculated as **H** = **X**(**X**′**X**)^−1^**X**′, in which **X** was a model matrix of the explanatory variables (i.e., location and stratum) constructed using the function model.matrix() in the *stats* package. Then, the matrix of fitted values is **HGH**, while that of residuals is (**I** − **H**)**G**(**I** − **H**), in which **I** is the identity matrix (McArdle & Anderson, 2001). We performed eigenvalue decomposition on (**I** − **H**)**G**(**I** − **H**), instead of on **G** as in Anderson & Thompson (2004), and scaled eigenvectors by square-root of respective eigenvalues in order to obtain principal coordinates of the residuals for each site. Locations of regional centroids in multivariate space were obtained by averaging the principal coordinates per year per region. For each region, Euclidean distances among centroids of different survey years were calculated from the averaged principal coordinates, which in turn were used to calculate *d*_*t*_ and ${d}_{t}^{b}$ (see Eqs. (4)–(7) in Anderson & Thompson, 2004 for details). For calculation of ${d}_{t}^{b}$, the first two years of data (2007 and 2009) were designated as a baseline in order to capture any gradual shifts in the assemblage structure since the establishment of Papahānaumokuākea Marine National Monument in 2006. Bootstrapping procedure followed the method described in Anderson & Thompson (2004), but treating the regional centroids as a population of sites. The procedure was repeated 1,000 times in order to obtain 50% and 95% confidence bounds for both *d*_*t*_ and ${d}_{t}^{b}$. If any regions had *d*_*t*_ and/or ${d}_{t}^{b}$ above the 95% confidence bounds at any time points, SIMPER analyses were done in order to identify which species typified each stratum in those particular years and compare them to those that typified each stratum in all other years.

## Results

In total, 268 species of reef fish were identified at 1,013 sites from the seven strata of the NWHI (Table 2). Many of these species were represented only by a few individuals at a small number of sites, and removing less-common species resulted in 84 species for data analyses. Average Bray–Curtis similarities of the structure of fish assemblages within each stratum group mostly ranged from 40 to 50 (possible range 0–100), although the stratum FRF-D had a lower average similarity of 35.8 (Table 3). The top eight species of fish that typified each stratum accounted for approximately 50% or more of the within-group similarities and were mostly herbivores and invertivores (Table 3). Overall, the invertivore *Thalassoma duperrey* was the most consistently abundant species in the NWHI having the highest average similarity contribution among all reef fish species in all but LAG-D stratum. *T. duperrey* also had the highest mean abundance in all of the strata within shallow or mid depths.

**Table 2:** Numbers of sites surveyed per stratum per year. Strata are backreef-shallow (BKR-S), forereef-shallow (FRF-S), forereef-mid (FRF-M), forereef-deep (FRF-D), lagoon-shallow (LAG-S), lagoon-mid (LAG-M) and lagoon-deep (LAG-D).

	2007	2009	2010	2011	2012	2014	2015	2016
BKR-S	9	10	10	5	4	7	4	0
FRF-S	9	21	14	26	5	15	18	22
FRF-M	29	73	43	41	34	42	41	89
FRF-D	16	43	19	42	20	25	33	60
LAG-S	22	27	13	20	10	0	0	6
LAG-M	27	4	15	4	15	0	0	5
LAG-D	5	1	4	3	3	0	0	0

**Table 3:** Top eight fish species that typified each stratum based on SIMPER analysis on the Bray–Curtis similarity calculated from square-root transformed abundance. The average similarity of each stratum is in parentheses.

Species	Av.Abund	Av.Sim	Sim/SD	%Contrib
**BKR-S (50.0)**				
*Thalassoma duperrey* (I)	3.60	10.54	3.54	21.08
*Stethojulis balteata* (I)	2.52	6.20	1.95	12.40
*Acanthurus triostegus* (H)	2.44	5.47	1.60	10.94
*Stegastes marginatus* (H)	2.61	5.30	1.36	10.59
*Coris venusta* (I)	1.82	4.19	1.11	8.38
*Thalassoma ballieui* (I)	1.07	2.00	0.96	4.00
*Macropharyngodon geoffroy* (I)	1.09	1.98	1.02	3.96
*Parupeneus multifasciatus* (I)	0.81	1.63	1.19	3.26
**FRF-S (41.4)**				
*Thalassoma duperrey* (I)	4.71	9.36	2.85	22.58
*Stegastes marginatus* (H)	2.53	3.84	1.38	9.27
*Acanthurus triostegus* (H)	2.57	3.70	1.37	8.94
*Thalassoma ballieui* (I)	1.19	2.09	1.52	5.03
*Acanthurus nigroris* (H)	1.52	2.01	0.99	4.86
*Chromis vanderbilti* (P)	2.62	1.89	0.53	4.55
*Chlorurus perspicillatus* (H)	1.21	1.80	1.08	4.34
*Stethojulis balteata* (I)	1.39	1.78	0.76	4.30
**FRF-M (44.4)**				
*Thalassoma duperrey* (I)	3.71	7.07	3.07	15.94
*Stegastes marginatus* (H)	2.13	2.94	1.06	6.63
*Acanthurus nigroris* (H)	1.56	2.15	1.19	4.85
*Parupeneus multifasciatus* (I)	1.27	2.13	1.70	4.81
*Chromis vanderbilti* (P)	2.36	2.13	0.63	4.81
*Ctenochaetus strigosus* (H)	1.58	1.89	0.89	4.26
*Bodianus albotaeniatus* (I)	1.00	1.86	2.06	4.20
*Acanthurus triostegus* (H)	1.50	1.71	0.97	3.86
**FRF-D (35.8)**				
*Thalassoma duperrey* (I)	2.07	3.63	1.35	10.12
*Centropyge potteri* (H)	2.02	3.36	1.33	9.38
*Chromis hanui* (P)	2.72	3.12	0.88	8.70
*Parupeneus multifasciatus* (I)	1.23	2.53	1.62	7.06
*Ctenochaetus strigosus* (H)	1.67	2.31	0.84	6.44
*Bodianus albotaeniatus* (I)	0.96	1.83	1.30	5.12
*Chromis ovalis* (P)	1.83	1.47	0.53	4.10
*Acanthurus nigroris* (H)	1.12	1.45	0.81	4.05
**LAG-S (42.9)**				
*Thalassoma duperrey* (I)	3.55	8.79	2.74	20.52
*Stethojulis balteata* (I)	1.95	4.38	1.59	10.21
*Stegastes marginatus* (H)	2.18	3.77	1.27	8.79
*Coris venusta* (I)	1.60	3.48	0.85	8.13
*Acanthurus triostegus* (H)	1.92	2.94	1.11	6.87
*Thalassoma ballieui* (I)	0.98	1.59	0.97	3.72
*Parupeneus multifasciatus* (I)	0.90	1.54	0.97	3.60
*Macropharyngodon geoffroy* (I)	0.87	1.51	0.80	3.51
**LAG-M (40.5)**				
*Thalassoma duperrey* (I)	2.50	5.89	2.90	14.55
*Chlorurus sordidus* (H)	2.00	3.45	1.16	8.52
*Scarus dubius* (H)	2.08	3.43	0.92	8.48
*Ctenochaetus strigosus* (H)	1.92	2.58	0.82	6.37
*Parupeneus multifasciatus* (I)	1.01	2.21	1.90	5.45
*Bodianus albotaeniatus* (I)	0.87	1.81	1.50	4.47
*Chaetodon miliaris* (P)	0.87	1.41	0.85	3.48
*Stegastes marginatus* (H)	1.20	1.40	0.61	3.46
**LAG-D (42.6)**				
*Ctenochaetus strigosus* (H)	3.32	6.34	1.28	14.88
*Chlorurus sordidus* (H)	1.96	3.49	1.05	8.18
*Scarus dubius* (H)	1.93	3.14	0.92	7.36
*Chromis hanui* (P)	1.92	3.00	1.16	7.03
*Centropyge potteri* (H)	1.54	2.91	1.48	6.84
*Parupeneus multifasciatus* (I)	1.12	2.74	2.02	6.43
*Thalassoma duperrey* (I)	1.59	2.74	1.24	6.43
*Bodianus albotaeniatus* (I)	0.99	2.39	1.62	5.62

**Notes.**

Trophic categories are in parentheses: H, herbivore; I, invertivore; P, planktivore. The heading of each column shows: “Av.Abund”, average square-root transformed abundance; “Av.Sim”, average similarity contribution; “Sim/SD”, ratio of the average similarity contribution to the standard deviation of similarity contribution; “%Contrib”, percentage of the contribution by the species to the within-group similarity.

The top eight typifying species were the same between the two shallow strata, LAG-S and BKR-S, and similar among all three shallow strata, with invertivores, *T. duperrey*, *Thalassoma ballieui* and *Stethojulis balteata* and herbivores, *Acanthurus triostegus* and *Stegastes marginatus*, being consistently present in high abundance. Within forereef habitat, there was a gradual species turnover with increasing depths, with invertivores, *Parupeneus multifasciatus* and *Bodianus albotaeniatus*, and an herbivore *Ctenochaetus strigosus* being consistently abundant in the stratum FRF-M, and planktivores, *Chromis hanui* and *Chromis ovalis*, and an herbivore *Centropyge potteri* in the stratum FRF-D. Species turnover was relatively similar in lagoon, with *C. strigosus*, *B. albotaeniatus*, *C. hanui* and *C. potteri* becoming more abundant with increasing depths, but two species of parrotfish *Chlorurus sordidus* and *Scarus dubius* were also consistently abundant at depths ≥6 m (LAG-M and LAG-D).

DISTLM conditional tests showed that the location variable explained a significant proportion (4.7%) of the variation in the structure of fish assemblages as a whole (*P* = 0.0002, Table 4). The stratum variable also explained significant, and the largest, proportion of the variation in the assemblage structure with an additional 13.8% (*P* = 0.0002, Table 4). Together these two variables explained 18.5% of the variation in the assemblage structure. The year variable added only 3.3% to the explained variation after the location and stratum variables were fitted. However, this was also statistically significant (*P* = 0.0002, Table 4) confirming the presence of temporal variability in the structure of fish assemblages after the effects of geographical locations and strata were taken into account.

**Table 4:** Results of DISTLM conditional tests for the structure of fish assemblages based on the Bray–Curtis measure. Explanatory variables used were location, stratum and year.

	Explained SS	Pseudo-*F*	*P*	Proportion	*R*^2^
Location	100,230	50.30	0.0002	0.047	0.047
+ Stratum	291,880	28.38	0.0002	0.138	0.185
+ Year	70,038	6.04	0.0002	0.033	0.218

**Table 5:** Numbers of sites surveyed per island/atoll per year.

		2007	2009	2010	2011	2012	2014	2015	2016
South	Nihoa	0	0	0	8	0	0	0	0
	Mokumanamana	0	13	0	8	0	0	0	0
	FFS	57	0	27	8	15	27	8	47
Mid	Gardner	0	0	0	12	0	0	0	0
	Maro	0	39	0	25	0	0	17	0
	Laysan	0	14	0	23	0	0	8	0
	Lisianski	8	19	25	9	25	28	18	40
North	PHA	52	0	41	18	31	0	23	56
	Midway	0	51	0	30	0	34	14	0
	Kure	0	43	25	0	20	0	8	39

**Figure 2:**
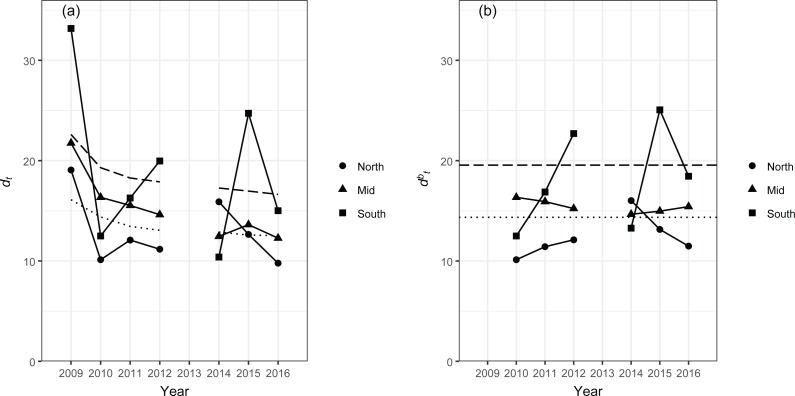
Distance-based multivariate control charts, *d*_*t*_ (a) and ${d}_{t}^{b}$ (b), for the south, mid and north regions of the NWHI, constructed based on DISTLM residuals, (**I** − **H**)**G**(**I** − **H**). The dashed line indicates the 95th percentile and the dotted line indicates the 50th percentile, both estimated from 1,000 bootstrap samples. For calculation of ${d}_{t}^{b}$, the first two years of data (2007 and 2009) were designated as a baseline.

The grouping of islands/atolls into the three regions, south, mid and north, resulted in consistent data points for each region for each time period, although the total numbers of survey sites were still relatively low in some years, particularly in the south region (Table 5). Multivariate control charts showed both *d*_*t*_ and ${d}_{t}^{b}$ above the 95% confidence bounds in the south region in 2012 and 2015 (Fig. 2). A high value of *d*_*t*_ was also observed in the south region in 2009, but this is likely an artifact as the *d*_*t*_ in 2009 was simply the distance between the centroids of observations in 2007 and those in 2009 (see Anderson & Thompson, 2004 for details). In the south region in 2012, identities of the top eight species that typified sites from two lagoon strata, LAG-M (seven sites) and LAG-D (three sites), were relatively similar to those that typified these strata in other years (Tables 6 and 7). On the other hand, two forereef strata, FRF-M (three sites) and FRF-D (two sites), had more different assemblages of typifying species compared to those in other years (Tables 6 and 7). Specifically, two invertivores, *Pseudojuloides cerasinus* and *Oxycheilinus bimaculatus*, which were not among the top eight typifying species for the entire NWHI (Table 3) or in all other years for the south region (Table 6), were consistently present in high abundance in the stratum FRF-M in 2012. Similarly, two butterflyfish *Chaetodon fremblii* and *Chaetodon trifascialis* and an herbivore *Zebrasoma flavescens* were consistently abundant in the stratum FRF-D in 2012 (Table 7), but these species were never among the top eight typifying species for the entire NWHI (Table 3) or in all other years for the south region (Table 6). There were no surveys from the shallow strata (i.e., BKR-S, FRF-S and LAG-S) in the south region in 2012.

**Table 6:** Top eight fish species that typified each stratum in the south region based on SIMPER analysis on the Bray–Curtis similarity calculated from square-root transformed abundance, excluding observations from 2012 and 2015. The average similarity of each stratum is in parentheses.

Species	Av.Abund	Av.Sim	Sim/SD	%Contrib
**FRF-S (36.26)**				
*Thalassoma duperrey* (I)	3.60	6.96	2.23	19.19
*Chromis vanderbilti* (P)	4.99	5.44	1.08	15.01
*Acanthurus nigrofuscus* (H)	2.99	3.95	1.19	10.89
*Plectroglyphidodon imparipennis* (I)	1.51	2.18	1.16	6.01
*Acanthurus triostegus* (H)	1.73	1.86	0.90	5.13
*Stegastes marginatus* (H)	1.17	1.60	1.15	4.42
*Coris venusta* (I)	1.14	1.37	0.33	3.78
*Acanthurus olivaceus* (H)	1.25	1.00	0.50	2.76
**FRF-M (43.24)**				
*Thalassoma duperrey* (I)	2.82	6.29	2.78	14.55
*Chromis vanderbilti* (P)	3.44	4.45	0.87	10.28
*Acanthurus nigrofuscus* (H)	1.89	2.85	1.17	6.58
*Parupeneus multifasciatus* (I)	1.24	2.55	2.15	5.90
*Bodianus bilunulatus* (I)	0.92	1.87	1.92	4.33
*Acanthurus olivaceus* (H)	1.29	1.85	0.94	4.29
*Paracirrhites arcatus* (I)	1.18	1.62	0.80	3.75
*Macropharyngodon geoffroy* (I)	1.04	1.60	1.01	3.69
**FRF-D (34.60)**				
*Thalassoma duperrey* (I)	1.62	2.93	1.42	8.47
*Centropyge potteri* (H)	1.71	2.78	1.13	8.03
*Chromis hanui* (P)	2.32	2.69	0.86	7.77
*Parupeneus multifasciatus* (I)	1.18	2.64	1.63	7.62
*Acanthurus nigrofuscus* (H)	1.61	2.32	1.09	6.71
*Ctenochaetus strigosus* (H)	1.82	2.13	0.72	6.16
*Sufflamen bursa* (I)	0.89	1.77	1.12	5.11
*Bodianus bilunulatus* (I)	0.86	1.72	1.27	4.97
**LAG-M (47.47)**				
*Ctenochaetus strigosus* (H)	2.94	5.77	1.58	12.17
*Thalassoma duperrey* (I)	2.02	4.92	4.25	10.36
*Chlorurus sordidus* (H)	2.32	4.64	1.92	9.78
*Scarus dubius* (H)	2.26	4.13	1.18	8.69
*Parupeneus multifasciatus* (I)	1.22	3.06	4.15	6.44
*Bodianus bilunulatus* (I)	1.02	2.42	2.85	5.10
*Acanthurus nigrofuscus* (H)	1.35	2.03	1.04	4.29
*Centropyge potteri* (H)	1.02	1.59	0.98	3.35
**LAG-D (39.04)**				
*Ctenochaetus strigosus* (H)	3.14	5.79	1.06	14.82
*Chlorurus sordidus* (H)	1.81	3.33	1.04	8.52
*Parupeneus multifasciatus* (I)	0.95	2.68	1.73	6.87
*Scarus dubius* (H)	1.73	2.61	0.72	6.69
*Centropyge potteri* (H)	1.21	2.50	1.26	6.40
*Chromis hanui* (P)	1.35	2.42	0.96	6.19
*Bodianus bilunulatus* (I)	0.88	2.30	1.36	5.89
*Thalassoma duperrey* (I)	1.17	2.21	1.02	5.66

**Notes.**

Trophic categories are in parentheses after species names: H, herbivore; I, invertivore; P, planktivore. The heading of each column shows: “Av.Abund”, average square-root transformed abundance; “Av.Sim”, average similarity contribution; “Sim/SD”, ratio of the average similarity contribution to the standard deviation of similarity contribution; “%Contrib”, percentage of the contribution by the species to the within-group similarity.

**Table 7:** Top eight fish species that typified each stratum in the south region in 2012 based on SIMPER analysis on the Bray–Curtis similarity calculated from square-root transformed abundance. The average similarity of each stratum is in parentheses. There were no surveys in the strata FRF-S and BKR-S in the south region in 2012.

Species	Av.Abund	Av.Sim	Sim/SD	%Contrib
**FRF-M (34.70)**				
*Thalassoma duperrey* (I)	1.92	4.34	5.48	12.51
*Parupeneus multifasciatus* (I)	1.72	4.24	2.00	12.23
*Chromis vanderbilti* (P)	2.35	4.08	3.98	11.74
*Chromis hanui* (P)	2.31	2.60	4.91	7.49
*Coris venusta* (I)	1.44	2.39	0.58	6.89
*Pseudojuloides cerasinus* (I)	1.20	2.23	0.58	6.42
*Oxycheilinus bimaculatus* (I)	1.26	2.14	0.58	6.17
*Bodianus bilunulatus* (I)	0.76	2.04	3.98	5.87
**FRF-D (40.97)**				
*Chromis hanui* (P)	2.63	5.19	–	12.67
*Bodianus bilunulatus* (I)	1.41	4.24	–	10.35
*Chaetodon fremblii* (I)	1.50	4.24	–	10.35
*Chlorurus sordidus* (H)	2.71	4.24	–	10.35
*Zebrasoma flavescens* (H)	1.93	4.24	–	10.35
*Ctenochaetus strigosus* (H)	2.64	3.67	–	8.96
*Thalassoma duperrey* (I)	1.48	3.67	–	8.96
*Chaetodon trifascialis* (C)	1.11	3.00	–	7.32
**LAG-M (53.20)**				
*Ctenochaetus strigosus* (H)	3.45	5.40	1.26	10.15
*Chlorurus sordidus* (H)	2.47	5.35	2.51	10.05
*Thalassoma duperrey* (I)	2.17	4.58	4.01	8.60
*Scarus dubius* (H)	1.71	3.04	1.39	5.72
*Acanthurus nigrofuscus* (H)	1.49	2.78	1.99	5.23
*Bodianus bilunulatus* (I)	1.14	2.77	5.80	5.20
*Chaetodon trifascialis* (C)	1.30	2.71	2.32	5.10
*Chaetodon fremblii* (I)	1.10	2.69	5.84	5.06
**LAG-D (65.36)**				
*Ctenochaetus strigosus* (H)	4.24	9.08	7.30	13.90
*Chlorurus sordidus* (H)	3.20	7.60	13.21	11.63
*Thalassoma duperrey* (I)	2.16	5.38	15.15	8.23
*Chromis hanui* (P)	2.84	5.03	2.17	7.70
*Scarus dubius* (H)	2.72	4.85	4.57	7.42
*Centropyge potteri* (H)	2.05	4.52	9.10	6.91
*Zebrasoma flavescens* (H)	2.26	4.44	8.07	6.79
*Parupeneus multifasciatus* (I)	1.60	3.40	6.34	5.21

**Notes.**

Trophic categories are in parentheses after species names: H, herbivore; I, invertivore; P, planktivore; C, Corallivore. The heading of each column shows: “Av.Abund”, average square-root transformed abundance; “Av.Sim”, average similarity contribution; “Sim/SD”, ratio of the average similarity contribution to the standard deviation of similarity contribution; “%Contrib”, percentage of the contribution by the species to the within-group similarity.

In the south region in 2015, fishes that typified sites from the forereef strata, FRF-S (two sites), FRF-M (three sites) and FRF-D (three sites), were very different from species that typified these strata in other years in terms of both species identities and abundances (Tables 6 and 8). While the identities of the top eight typifying species were more similar between 2015 and other years at depths <6 m (FRF-S) than at depths ≥6 m, abundance of a planktivore *Chromis vanderbilti* was, on average, higher for 2015 than other years, with mean square-root transformed abundances of 6.23 and 4.99, respectively (Tables 6 and 8). Increased abundances of small-bodied planktivores were also observed at 6–18 m depths (FRF-M), with *C. vanderbilti*, *C. hanui* and *Chromis acares* being the most numerically abundant species (Table 8). At 18–30 m depths (FRF-D), *C. hanui* was consistently abundant in 2015, but the mean square-root transformed abundance was 0.94 and relatively low (Table 8); this fish had the mean square-root transformed abundance of 2.32 and was the most numerically abundant species in this stratum in other years (Table 6). In addition, similar to the stratum FRF-M in 2012, two invertivores *P. cerasinus* and *O. bimaculatus* were consistently present in high abundance in the stratum FRF-D in 2015. There were no surveys from either the backreef or lagoon stratum in the south region in 2015.

**Table 8:** Top eight fish species that typified each stratum in the south region in 2015 based on SIMPER analysis on the Bray–Curtis similarity calculated from square-root transformed abundance. The average similarity of each stratum is in parentheses. There were no surveys in the stratum BKR-S and lagoon habitat in the south region in 2015.

Species	Av.Abund	Av.Sim	Sim/SD	%Contrib
**FRF-S (52.89)**				
*Chromis vanderbilti* (P)	6.32	9.98	–	18.87
*Thalassoma duperrey* (I)	4.68	7.27	–	13.75
*Paracirrhites arcatus* (I)	2.68	5.29	–	10.01
*Acanthurus nigrofuscus* (H)	2.16	4.67	–	8.82
*Acanthurus olivaceus* (H)	1.87	4.32	–	8.17
*Plectroglyphidodon johnstonianus* (C)	1.87	4.32	–	8.17
*Acanthurus triostegus* (H)	1.11	2.49	–	4.72
*Macropharyngodon geoffroy* (I)	1.56	2.49	–	4.72
**FRF-M (36.42)**				
*Chromis vanderbilti* (P)	5.63	8.13	3.01	22.33
*Chromis hanui* (P)	5.24	4.08	1.65	11.20
*Chromis acares* (P)	5.67	3.91	0.58	10.73
*Macropharyngodon geoffroy* (I)	3.01	3.70	3.77	10.17
*Parupeneus multifasciatus* (I)	1.98	3.33	5.97	9.13
*Sufflamen bursa* (I)	1.82	3.24	3.80	8.90
*Thalassoma duperrey* (I)	2.37	1.61	0.58	4.41
*Acanthurus triostegus* (H)	1.62	0.91	0.58	2.49
**FRF-D (32.75)**				
*Oxycheilinus bimaculatus* (I)	3.65	9.60	2.29	29.31
*Parupeneus multifasciatus* (I)	1.98	4.33	1.79	13.22
*Sufflamen fraenatum* (I)	1.00	3.83	6.91	11.68
*Pseudojuloides cerasinus* (I)	1.51	3.63	2.99	11.10
*Centropyge fisheri* (H)	2.16	2.49	0.58	7.61
*Acanthurus olivaceus* (H)	2.02	2.13	0.58	6.49
*Chaetodon kleinii* (P)	1.77	2.13	0.58	6.49
*Chromis hanui* (P)	0.94	1.50	0.58	4.59

**Notes.**

Trophic categories are in parentheses after species names: H, herbivore; I, invertivore; P, planktivore; C, Corallivore. The heading of each column shows: “Av.Abund”, average square-root transformed abundance; “Av.Sim”, average similarity contribution; “Sim/SD”, ratio of the average similarity contribution to the standard deviation of similarity contribution; “%Contrib”, percentage of the contribution by the species to the within-group similarity.

## Discussion

The structure of fish assemblages on shallow (≤30 m) reefs of the NWHI was affected by both geographical location and habitat types. Despite the long distance of ∼2,000 km from the southeastern to the northwestern end of the NWHI and the latitudinal gradient that affects seawater temperature and, in turn, the structure of fish assemblages (Mundy, 2005), the variation in the assemblage structure explained by these two variables was much larger for the habitat (i.e., stratum) variable (Table 4). Changes in the structure of fish assemblages along depth were also evident for both forereef and lagoon habitats when species that typified the strata across the NWHI were identified in SIMPER analysis (Table 3). The effect of habitat types on coral reef fish assemblages, potentially resulting from differences in water movement energy, have been previously described (McGehee, 1994). Differences in fish assemblages between barrier and patch reef habitats have also been reported from FFS and Midway in the NWHI (DeMartini, Parrish & Parrish, 1996). The present study confirms the importance of including habitat types and depth in the survey design of fish monitoring in the NWHI and accounting for their effects when analyzing the data.

On shallow-water reefs of the NWHI, wrasses, particularly *Thalassoma duperrey*, were the most consistently abundant taxon; they accounted for all invertivores that typified each stratum, with an exception of the goatfish *Parupeneus multifasciatus* (Table 3). In lagoon habitat, six out of the top eight typifying species were wrasses at 0–6 m depths, but they became less abundant at deeper depths, being replaced by *P. multifasciatus* and herbivorous surgeonfish and parrotfish at 6–18 m and then by *Centropyge potteri* and *Chromis hanui* at 18–30 m depths. In forereef habitat, herbivorous surgeonfish and damselfish were consistently abundant in addition to invertivores at 0–18 m depths, but similar to lagoon habitat, *C. potteri* and *C. hanui* also became abundant at 18–30 m depths. Overall numerical abundance of herbivores and invertivores on shallow reefs is consistent with the results of a previous study in the NWHI that investigated changes in the trophic structure of fish assemblages from euphotic (1–30 m) to mesophotic (>30 m) depths (Fukunaga et al., 2016). In that study, *C. potteri* and *C. hanui* were two of the top three species that typified 27–40 m depths. Therefore, these two species seem to characterize lower euphotic and upper mesophotic depths in the NWHI.

Herbivory plays an important role in shaping benthic communities on shallow-water coral reefs by regulating algal biomass (McCook, 1999). In the main Hawaiian Island of O‘ahu, the territorial damselfish, *Stegastes marginatus* (previously referred to as *S. fasciolatus*, see Randall, 2007), has been documented to defend a small patch of the bottom (∼1 m^2^) and to graze algal mats inside, while parrotfish and surgeonfish grazing all erect algae outside the territory (Hixon & Brostoff, 1996). In the present study, the damselfish *S. marginatus* was consistently abundant in all the strata within 0–18 m depth (Table 3). Herbivores that typified these strata also included surgeonfish (*Acanthurus triostegus*, *Acanthurus nigroris*, *Ctenochaetus strigosus*), parrotfish (*Chlorurus perspicillatus*, *Chlorurus sordidus*, *Scarus dubius*) or both (Table 3). Thus, the same grazing pattern could potentially exist in the NWHI at <18 m depths where *S. marginatus*, surgeonfish and parrotfish were consistently abundant. At greater depths, however, *S. marginatus* was not numerically abundant in either the forereef habitat or lagoon; herbivores were numerically dominated by the angelfish *C. potteri* and parrotfish in the FRF-D and LAG-D strata, respectively.

While the presence of temporal variability in the structure of reef fish assemblages can be statistically tested through multivariate analyses such as analysis of similarity (Clarke & Warwick, 2001), permutational multivariate analysis of variance (Anderson, 2001; McArdle & Anderson, 2001) and DISTLM, multivariate control charts pinpoint when a change in the assemblage structure is more than what would be expected from natural temporal variability (Anderson & Thompson, 2004). In the present study, *d*_*t*_ and ${d}_{t}^{b}$ in the south region in 2012 and 2015 were both above the control charts’ 95% confidence bounds (Fig. 2). Examining species that typified each stratum in these years and comparing them with those in other years using SIMPER analyses revealed how fish assemblages differed in these two years; the increases in *d*_*t*_ and ${d}_{t}^{b}$ in both 2012 and 2015 were due to changes in relative abundances of fish species. The species identified to typify each stratum in these two years included *Oxycheilinus bimaculatus*, *Chaetodon fremblii* and *Zebrasoma flavescens* that have previously been reported to be relatively common in shallow reef habitats in the NWHI (DeMartini, Parrish & Parrish, 1996; Parrish & Boland, 2004). Changes in the structure of fish assemblages in 2015 were also partly due to increases in abundances of small-bodied planktivores (*Chromis vanderbilti*, *Chromis acares* and *C. hanui*) in forereef habitat at 0–18 m depths and a decrease in abundance of *C. hanui* at 18–30 m depths. More importantly, the typifying species of these two years did not include any introduced species, thus there was no evidence of introduced species displacing native fishes.

**Figure 3:**
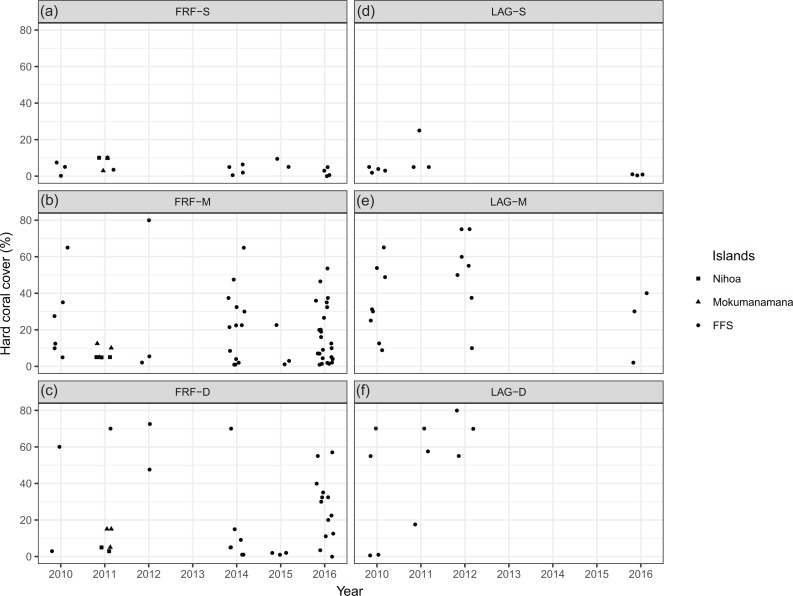
Percent cover of hard corals at each survey site in the south region: Nihoa, Mokumanamana and FFS. Horizontal jitters were added for overlapping values.

There are some important limitations on the interpretation of multivariate control charts in the present study where surveys were performed at different sites every year. Fish monitoring surveys can be accompanied by habitat surveys to record variables such as benthic composition (Floeter et al., 2007) and habitat complexity (Gratwicke & Speight, 2005) that are known to influence fish assemblages. For surveys using permanent transects, differences in fish assemblages or habitat likely reflect actual changes in these variables over time at each site. On the other hand, observations based on stratified random sampling are dependent on site selection at each time point, and changes in fish assemblages or habitat variables are not representative of any specific sites; multivariate control charts identify changes in the multivariate centroids (or averages) of response variables for each group of sites (i.e., region in our case). Interpretation of temporal changes becomes particularly difficult when the number of sites surveyed from each stratum is small, as is the case in the present study in the south region in 2012 and 2015. While differences in habitat or any other environmental variables may be directly or indirectly linked to unusual observations of fish assemblages identified by control charts, such differences could be either actual changes in the habitat over time or simply results of site selection where a small number of “unusual” sites happened to be chosen. In the present study, although habitat variables were not collected consistently over time, percent covers of hard corals (recorded since 2010) were relatively low at all of the survey sites in the strata FRF-M and FRF-D in the south region in 2015 (Fig. 3). In particular, all three sites surveyed in the stratum FRF-D had ≤5% coral covers and their reef types were recorded as rubble. Similarly, two of the three sites surveyed in the stratum FRF-M in the south region in 2012 also had a reef type of pavement with ≤5% coral covers. These were the strata where two invertivores *Pseudojuloides cerasinus* and *O. bimaculatus* were consistently abundant. Therefore, the alarm triggered by the control charts could be, at least in part, due to the small numbers of survey sites and low coral covers at these sites, particularly in 2015.

**Figure 4:**
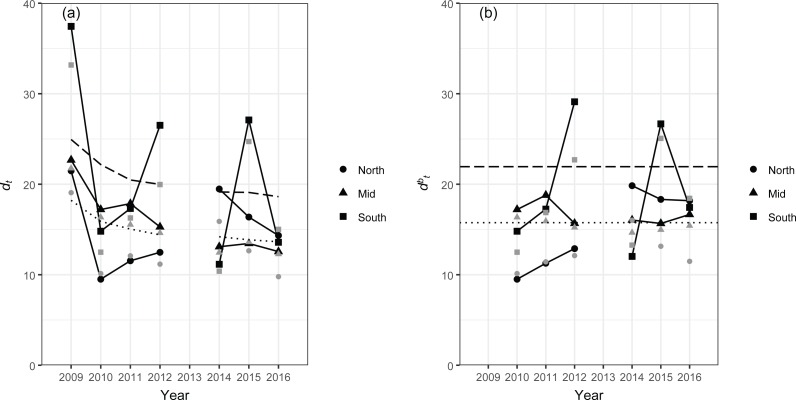
Distance-based multivariate control charts, *d*_*t*_ (a) and ${d}_{t}^{b}$ (b), for the south, mid and north regions of the NWHI, constructed based on the original data matrix, **G**. The dashed line indicates the 95th percentile and the dotted line indicates the 50th percentile, both estimated from 1,000 bootstrap samples. For calculation of ${d}_{t}^{b}$, the first two years of data (2007 and 2009) were designated as a baseline. Values of *d*_*t*_ and ${d}_{t}^{b}$ after DISTLM (as shown in Fig. 2) are overlaid in gray for comparison. Note that the 95th percentile lines are specific to the *d*_*t*_ and ${d}_{t}^{b}$ constructed based on the original data matrix **G,** so they do not apply to the *d*_*t*_ and ${d}_{t}^{b}$ after DISTLM shown in gray.

Despite the relatively small proportion of variation in the structure of fish assemblages explained by the location and stratum variables (<20%, Table 4), the use of DISTLM residuals, rather than the original data matrix, made some differences to the control charts when compared to those constructed using the original data matrix (Fig. 4). For example, values of *d*_*t*_ and ${d}_{t}^{b}$ for the south region in 2012 were relatively comparable to and higher than, respectively, those in 2015 when the original data matrix was used (Fig. 4). On the other hand, the use of DISTLM residuals considerably reduced both *d*_*t*_ and ${d}_{t}^{b}$ in 2012 resulting in these values being lower than those in 2015 (Fig. 2). This is consistent with the results of SIMPER analysis; when comparing the top eight species that typified each stratum in the south region in 2012 (Table 7) and 2015 (Table 8), those in 2012 are more similar to the eight typifying species from all other years (Table 6). Our use of DISTLM to obtain residuals for multivariate control charts is parallel to the concept of detrending, in which a temporal or spatial trend in ecological data is extracted prior to data analyses (Legendre & Legendre, 2012). Here, we limited explanatory variables for DISTLM to those that were part of our survey design (i.e., strata) and a potential source of spatial correlation (i.e., geographical location). While it is possible to include other explanatory variables, such as reef types and coral covers, for detrending, successively extracting multiple trends requires some caution as each extraction distorts the residuals (Legendre & Legendre, 2012).

For monitoring of a protected area, BACI (Before-After-Control-Impact) designs are often used to compare biological communities inside and outside the protected area over time, but lack of comparable sites can preclude such a monitoring design while scientists and managers try to evaluate whether the protected area is achieving its objectives (Stringell et al., 2013). Multivariate control charts allow for evaluation of protected areas even in the absence of appropriate control sites (Anderson & Thompson, 2004; Stringell et al., 2013). The present study showed that multivariate control charts can be extended to a stratified random sampling design and used to evaluate the status of biological communities in a very large protected area. Here, we focused our analyses on non-transient, resident reef fish species in the NWHI. The use of the Bray–Curtis measure for multivariate control charts followed by SIMPER analyses to identify typifying species for each stratum put more emphasis on common species, so less-common species were removed prior to the analyses. Each user should adjust, however, pre-treatments of data (e.g., data transformation and removal of rare species), choice of dissimilarity/distance measures and methods of investigation following multivariate control charts according to the nature of their study and ecological questions they have.

In conclusion, reef fish assemblages in the NWHI were mostly stable, with exceptions in the south region (Nihoa, Mokumanamana and FFS) in 2012 and 2015. The potential sensitivity of multivariate control charts to a small sample size combined with low coral cover when applied to stratified random sampling calls for some caution during the process of survey site selection where selected sites should cover a wide range of coral reef habitat representative of the NWHI. In addition, multivariate control charts do not provide understanding of the causes, mechanisms or ecological processes of observed changes in biological communities. Therefore, for ecological monitoring of the Papahānaumokuākea Marine National Monument, it is critical for researchers and managers to identify potential threats to shallow reef habitats of the NWHI and ensure that future efforts of fish monitoring are accompanied by measures of specific environmental variables that can be used to assess whether changes in fish assemblages are associated with any of these threats.

##  Supplemental Information

10.7717/peerj.3651/supp-1 Data S1 R scripts for multivariate control chars combined with a distance based linear model R scripts for obtaining multivariate control charts are provided in the supplemental file, S1.R. Raw data are also included as spe_mcc.Rda for species data and as fac_mcc.Rda for explanatory variables.

## Additional Information and Declarations

### Field Study Permissions

The following information was supplied relating to field study approvals (i.e., approving body and any reference numbers): Field work in the Papahānaumokuākea Marine National Monument (Northwestern Hawaiian Islands) was conducted under Papahānaumokuākea Marine National Monument research permits.
